# Photoperiod induced the pituitary differential regulation of lncRNAs and mRNAs related to reproduction in sheep

**DOI:** 10.7717/peerj.10953

**Published:** 2021-04-21

**Authors:** Xiaoyun He, Lin Tao, Yingjie Zhong, Ran Di, Qing Xia, Xiangyu Wang, Xiaofei Guo, Shangquan Gan, Xiaosheng Zhang, Jinlong Zhang, Qiuyue Liu, Mingxing Chu

**Affiliations:** 1 Institute of Animal Sciences, Chinese Academy of Agricultural Sciences, Beijing, China; 2 Tianjin Institute of Animal Sciences, Tianjin, China; 3 Xinjiang Academy of Agricultural and Reclamation Sciences, Xinjiang, China

**Keywords:** Sheep, Pituitary, RNA sequencing, Photoperiod, lncRNA, mRNA

## Abstract

The pituitary is a vital endocrine organ that regulates animal seasonal reproduction by controlling the synthesis and secretion of the hormone. The change of photoperiod is the key factor affecting the function of the pituitary in animals, but the mechanism is unclear. Here, we studied the transcriptomic variation in pars distalis (PD) of the pituitary between short photoperiod (SP) and long photoperiod (LP) using RNA sequencing based on the OVX+E_2_ sheep. 346 differentially expressed (DE) lncRNAs and 186 DE-mRNA were found in the PD. Moreover, function annotation analysis indicated that the reproductive hormones and photoperiod response-related pathways including aldosterone synthesis and secretion, insulin secretion, thyroid hormone synthesis, and circadian entrainment were enriched. The interaction analysis of mRNA-lncRNA suggested that MSTRG.240648, MSTRG.85500, MSTRG.32448, and MSTRG.304959 targeted *CREB3L1* and *DUSP6*, which may be involved in the photoperiodic regulation of the PD. These findings provide resources for further study on the seasonal reproductive in ewes.

## Introduction

Animals can schedule reproduction events to maximize adaptation to the changing environment and the survival of offspring. Most animals, including birds, mammals and even in the human, have a highly accurate mechanism for photoperiod measurement and show dramatic changes in seasonal response to small changes in photoperiod (Nakayama & Yoshimura, 2017; Guh, Tamai & Yoshimura, 2019). In sheep, the efficiency of reproduction is significantly related to the frequency of estrus (Li et al., 2019a). However, photoperiodic seasonal change is the main inducement that affects female mammalian seasonal estrus and reproduction. Compared to ewes within estrous cycling (short photoperiod, SP) with anestrus (long photoperiod, LP), early studies showed that there are significant physiological and neuroendocrine differences in pituitary (Mcbride et al., 2012). It is well known that the start and stop of mammalian reproductive activities are controlled by hypothalamic-pituitary-gonadal axis (HPGA), and several reproductive hormones such as follicle-stimulating hormone (FSH), luteinizing hormone (LH), prolactin (PRL) are all secreted by PD of the pituitary gland (Nestor et al., 2018; Lincoln et al., 2006; Dardente et al., 2019), importantly, these hormones vary with the seasons or photoperiod in birds, goats and sheep (Nakayama & Yoshimura, 2017; Guh, Tamai & Yoshimura, 2019; Di et al., 2014; Zhai et al., 2018; Li et al., 2019a).

Remarkably, most of the reproductive hormones are proteins, non-coding RNA should be taken into account transcriptional regulation of their synthesis and secretion. LncRNA play a vital role in regulating the mammalian reproduction by involving in a series of biological processes such as gametogenesis (Mercer et al., 2010; Arun et al., 2012), placentation (Gao et al., 2012), reproductive hormone responses (Gao et al., 2012; Liu et al., 2018a). In our recent studies, we found that several differentially expressed lncRNAs (DE-lncRNA) in the hypothalamus and uterus may be involved in the sheep reproductive process by interacting with target genes in Small Tail Han sheep (Lai et al., 2012; Zhang et al., 2019). Moreover, Zheng et al. (2019) found that the target genes of the DE lncRNAs were significantly enriched in pituitary function and hormone-related pathways, which may participate in the ovine prolificacy. However, the studies on the expression pattern and potential roles of lncRNAs in the pituitary are still limited compared to the miRNAs, lncRNAs or mRNAs. The pituitary is an important endocrine gland that plays a connecting role in the hypothalamus and gonad. However, the work about the systematic analysis of expression pattern of lncRNAs in the key area of pituitary gland (such as PD) during the different photoperiod has not been performed.

To date, our understanding of photoperiod induced molecular neuroendocrine is still limited. In this study, to identify the role of lncRNAs and mRNAs in the pituitary associated with sheep reproduction based on different photoperiod treatments, high-throughput sequencing was performed on the PD of pitiutary to screen DE genes and DE lncRNAs, subsequently, bioinformatics analysis was used to identify the key lncRNAs that regulate the pituitary function and photoperiodic response in the PD. This study expands the understanding and catalogue of PD lncRNA in sheep, and provides candidate regulators of sheep reproduction regulation at the transcriptional level.

## Material and Methods

### Animal treatments and sample collection

The six Sunite ewes (35–40 kg, 3 years-old, clinically normal and non-pregnant) were selected and housed in the Tianjin Institute of Animal Sciences, Tianjin (39°N latitude), China. All ewes were raised under the same conditions, fed ad libitum and had free access to water. Before the experiment, the 6 Sunite ewes were ovariectomized (OVX) via midventral laparotomy the surgical procedures are as follows: firstly, we fixed the sheep on the frame to make the breasts fully exposed, and washed the stains around the abdomen with hot soapy water, then we scraped the wool cleanly. The surgical instruments and the hands of the surgeon were routinely disinfected. After the surgery, the ewes were allowed to recover for at least 30 days before hormone treatments. 30 days after arrival, 6 ewes were treated with Estradiol as described in the previous studies (Smith et al., 2007). Finally, 6 ewes were maintained in two photoperiod-controlled room (Room1: SP, Short Photoperiod, 8/16 h light-black; Room2: LP, Long Photoperiod, 8/16 h light-black). The slaughter was scheduled for SP42 day and LP42 day, Pituitary was rapidly removed from the brain, the PD was quickly separated,frozen in liquid nitrogen, and stored at −80 °C for subsequent study.

All the experimental procedures were approved by the Science Research Department of the Institute of Animal Sciences, Chinese Academy of Agricultural Sciences (IAS-CAAS) (Beijing, China). And ethical approval was given by the Animal Ethics Committee of the IAS (IAS2018-3).

### RNA Extraction and sequencing

Total RNA from each sample was isolated using the TRIzol reagent (Invitrogen, Carlsbad, CA, USA). RNA purity, concentration, and integrity were also detected following our previous method (La et al., 2019). Subsequently, sequencing libraries were generated using the rRNA-depleted RNA by NEBNext^®^ Ultra™ Directional RNA Library Prep Kit for Illumina^®^ (NEB, Ipswich, MA, USA). Finally, the libraries were sequenced on an Illumina platform.

### Sequence analysis

*Ovis aries* reference genome and gene annotation files were downloaded from the genome website directly (Oar_v4.0, https://www.ncbi.nlm.nih.gov/assembly/GCF_000298735.2). Clean data were obtained by removing reads containing adapter, ploy-N, and low quality reads were also removed from raw data. HISAT2 was used to map the clean reads to the reference genome, the software was run with ‘–rna-strandness RF’, and other parameters were set as default. The mapped reads of each sample were assembled by StringTie following the reference-based approach (Pertea et al., 2016). The assembled transcripts were annotated using the gffcompare program. Transcripts with length more than 200 nt and two exons were selected as lncRNA candidates. CPC, CNCI, Pfam, CPAT were used to distinguish the protein-coding genes from the non-coding RNAs. After the above four analyses, the remaining lncRNAs were used for subsequent analysis.

### Differential expression genes identification and qPCR validation

FPKM was used to measure the expression levels of transcripts. HTSeq was used to calculate the FPKM of both lncRNAs and mRNAs in each sample based on the Python, the program was run with ‘-igene_id -f bam –s reverse -a 10 –q’. A criterion of absolute log2 (fold change)>1 and *P* < 0.01 was used to identify differentially expressed genes using‘MARS’ of the Deseq. RNA sequencing results were validated by qPCR, in which 5 mRNAs and 5 lncRNAs were randomly selected, the information of primers were listed in Table 1. The cDNA synthesis and qPCR was performed as described in our previous study (Zhang et al., 2019). All samples were examined in triplicate. Relative expression levels of differentially expressed mRNAs and lncRNAs were normalized by *β*-actin using 2 ^−ΔΔCt^ method (Livak & Schmittgen, 2000).

**Table 1:** The specific primers for qPCR.

Transcript type	Transcript name	Forward primer	Reverse primer	Product size (bp)
mRNA	DUSP6	CTGGAACGAGAATACTGGCG	ATCTTCCAGGTAGAACGCCC	91
	ANGPTL2	AACTGTGCCCACTACCAGAA	ATCACCACCTTCTTGAGCGA	164
	SPRY4	CGTTGGTGCAGTGGTAGAAG	CGTTGGTGCAGTGGTAGAAG	152
	GHRH	CAGCAGGGAGAGAGAAACCA	CCAAGATGCTCTCCAATGCC	106
	FOSL2	TGCAGAAGGAGATCGCTGAA	CAGGACTGATCTTGCACACG	90
LncRNA	MSTRG.251736	ATCAGCATTCCCCTCTCTGG	CACAGACTGTTCTTTGCCCC	147
	MSTRG.202202	ATCAGCTCTCCCAGTCACAC	GCCTCCCTCTCTGAAAACCT	136
	MSTRG.26775	ACAAGGAGCTGATGTCACCA	TCAGGGTGGAGAGTGAGGTA	155
	MSTRG.130282	TGGTTTTCATTCTGCCTGCC	GATTTACTGTGCATGGCCCC	127
	MSTRG.50976	AACCCTAACCAGAGATGGCC	TCTAGAGGCTGTTTCTGGGC	93

### Functional annotation and enrichment analysis

The *cis* and *trans* functions of lncRNA were defined in our previous study (Zhang et al., 2019). The R packages, such as KOBAS and Goseq, were used to implement GO and KEGG enrichment analysis (Zheng et al., 2019). a corrected *P* < 0.05 (*q* < 0.05) were considered significantly enriched for GO and KEGG enrichment.

### Construction of mRNA–mRNA and lncRNA–mRNA networks

We select the mRNAs with High Spearman correlation Coefficient (*P* ≥ 0.9) as the *trans*-targets of DE-lncRNA, and the mRNA with distance less than 50 kb were selected as the Cis-targets of DE-lncRNA. To further explore the interactions between the DE-lncRNAs, target genes and DE-mRNAs in PD, The interaction networks of transcripts associated with pituitary function and reproduction were built using the DE-lncRNA, target genes of lncRNAs and DE-mRNAs. Visualization was achieved through software called Cytoscape (V3.6.1) (La et al., 2019; Zheng et al., 2019; Zhang et al., 2019).

### Statistical analysis

Student’s *T*-test of SPSS 20.0 statistical software were used to evaluate the experimental results . All data were shown as means with standard error (SE). one-way ANOVA was used for qPCR validation. *P* < 0.05 was considered statistically significant.

## Results

### Summary of sequencing data in ovine PD

In this study, 6 libraries were established to identify the changes in sheep PD transcriptome in both SP and LP. A total of 94.48G clean bases were obtained. The percentage of clean Q30 is more than 92.64%, and the ratio of clean reads and total mapped reads was larger than 95.29% and 93.57% respectively (Table 2).

**Table 2:** Summary information on the sequencing data.

Sample	Raw reads number	Clean reads number	Clean reads rate (%)	Clean bases (G)	Clean Q30 bases rate (%)	Total mapping rate (%)
SP42P1	116,206,488	113,856,274	97.98	15.91	93.79	94.29
SP42P2	117,226,392	112,967,194	96.37	15.78	92.64	93.57
SP42P3	129,006,964	125,514,778	97.29	17.53	94.03	94.23
LP42P1	111,936,010	107,803,564	96.31	15.06	94.11	95.21
LP42P2	116,237,926	110,762,742	95.29	15.47	94.02	93.39
LP42P3	106,513,748	103,666,030	97.33	14.48	93.99	94.12

### Identification of lncRNAs and mRNAs in ovine PD

**Figure 1:**
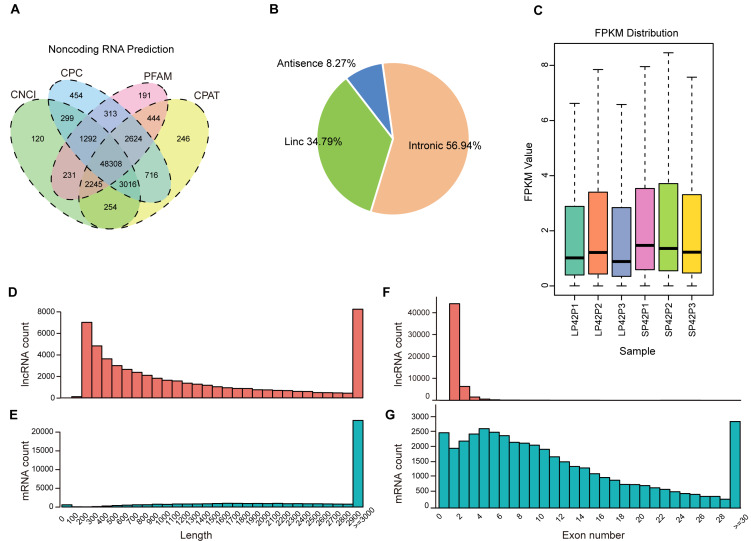
Identification of lncRNAs and mRNAs in ovine PD. (A–B) The screen of lncRNAs. (C) Boxplot of FPKM distribution of each sample. (D–E) The length statistics of lncRNA and mRNA. (F–G) The statistics of lncRNA and mRNA exon number.

After mapping to the sheep genome (Oar_v4.0), 48308 novel lncRNA were identified (Fig. 1A), in which the maximum proportion of intronic lncRNAs was 56.94%, followed by lincRNAs and antisense lncRNAs for a minimum percentage (Fig. 1B). The expression of transcripts in SP is higher than LP (Fig. 1C). Moreover, length between lncRNA and mRNA is similar in the PD (Figs. 1D–1E), the exon number of lncRNA is less than mRNA and most of which have 2 or 3 exons (Figs. 1F–1G).

**Figure 2:**
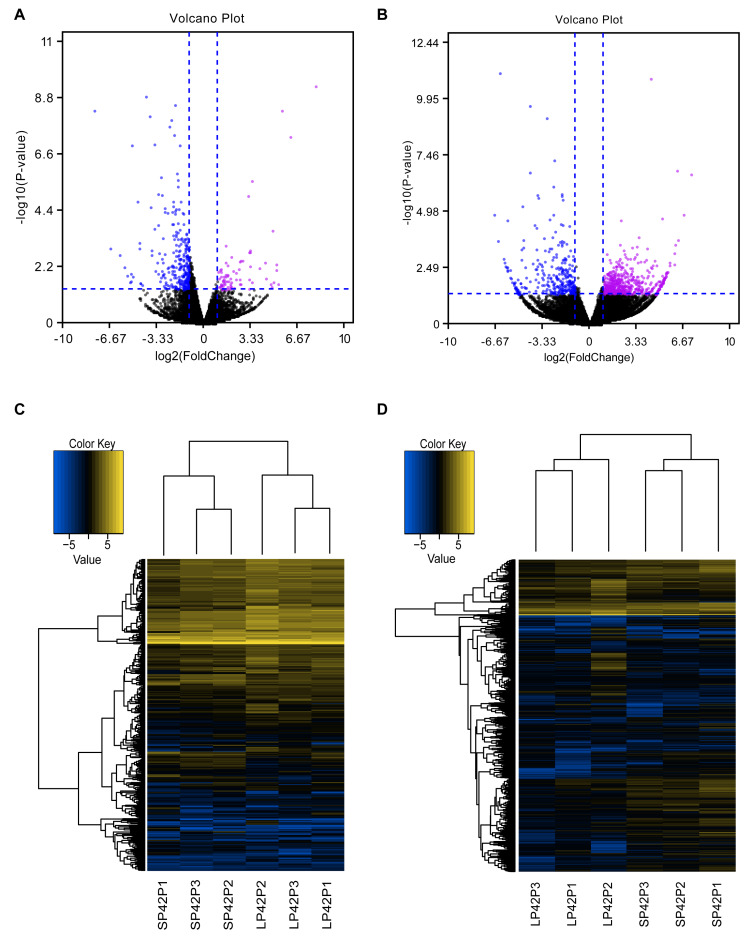
Analysis of differentially expressed transcripts. (A) Volcano map of differentially expressed mRNA in SP42 and LP42. (B) Volcano map of differentially expressed lncRNA in SP42 and LP42. (C) Hierarchical cluster analysis of DE-mRNA in SP42 and LP42. (D) Hierarchical cluster analysis of DE-lncRNA in SP42 and LP42. The logarithm base 2 was taken to calculate Euclidean distance according to the expression of DE-mRNA and DE-lncRNA in each sample, then Hierarchical cluster maps were obtained.Note: purple and blue represent up-regulated and down-regulated transcripts respectively.

### Analysis and verification of DE-lncRNAs and DE-mRNAs of ovine PD

**Figure 3:**
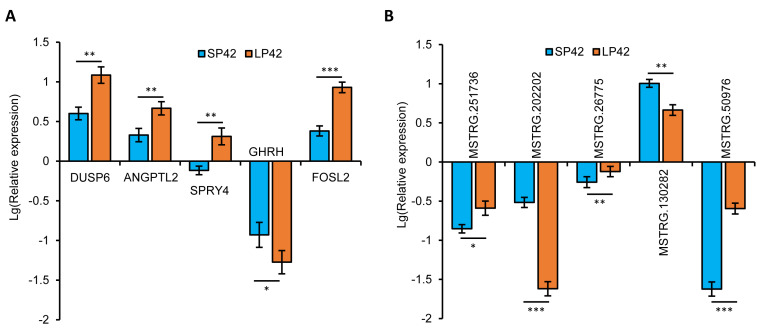
Validation of the expression patterns of lncRNA and mRNA using qRT-PCR. (A) The qPCR verification of the 5 DE-mRNAs in SP42 and LP42. (B) The qPCR verification of the 5 DE-lncRNAs in SP42 and LP42. The expression of transcripts was normalized by *β*-actin to determine relative expression using 2^−ΔΔCt^ method. Results were expressed as mean±SE, * represents *P* < 0.05, ** represents *P* < 0.01, *** represents *P* < 0.001.

In total, we identified 346 DE-lncRNA (Up-regulation 186 and down-regulation 163) and 186 DE-mRNA (Up-regulation 30 and down-regulation 156) comparing the SP42 to LP42 (Figs. 2A, 2B), the details of the up and down information can be found in Table S1 and Table S2. Besides, the hierarchical cluster analysis was performed to test the grouping is reasonable using DE-lncRNA and DE-mRNA (Figs. 2C, 2D). To verify the accuracy of sequencing, 5 DE-lncRNAs (MSTRG.251736, MSTRG.202202, MSTRG.26775, MSTRG.130282, MSTRG.50976) and 5 DE-mRNAs (*DUSP6*, *ANGPTL2*, *SPRY4*, *GHRH*, *FOSL2*) were selected randomly to detect the relative expression level in the SP42 and LP42 groups using qPCR. The expression levels of the 5 lncRNAs and 5 mRNAs were shown in Fig. 3 using the Lg(Relative expression), which were consistent with the RNA-seq both in lncRNA and mRNA.

### GO and KEGG enrichment analysis of DE-mRNAs and DE-lncRNAs

**Figure 4:**
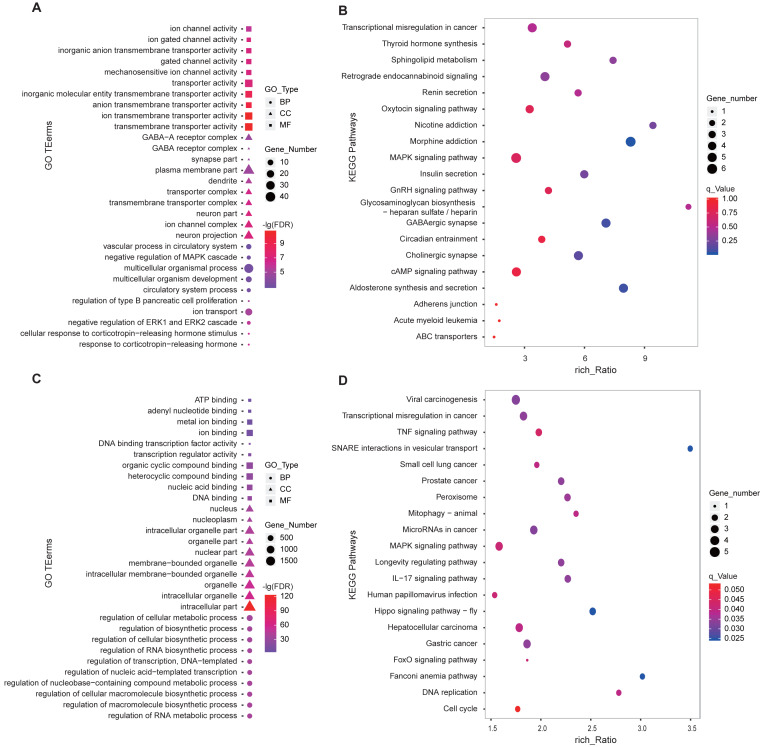
GO and KEGG enrichment analysis of the DE-lncRNAs and DE-mRNAs in PD. (A) GO function analysis of DE-mRNAs using the top 10 GO terms in each of BP, CC and MF. (B) Top 20 KEGG enrichment pathways of DE-mRNAs in ovine PD. (C) GO enrichment of DE-lncRNA targets using the top 10 GO terms in each of BP, CC and MF. (D) Top 20 KEGG enrichment pathways of DE-lncRNA targets in ovine PD. BP: Biological Process, CC: Cellular Component, MF: Molecular Function.

We performed GO and KEGG enrichment analysis using the 346 DE-lncRNA and 186 DE-mRNA. The most significant (FDR < 0.05) enriched the top 10 terms of each GO type were shown in Fig. 4. For DE-mRNA, the significant enriched GO terms were involved in reproduction and PD function including response to the corticotropin-releasing hormone, negative regulation of ERK1, and ERK2 cascade, ion transport, regulation of type B pancreatic cell proliferation (Fig. 4A). For DE-lncRNA, we used their targets to conduct GO enrichment and most of the significant enriched GO terms participate in the regulation of biological and cellular processes (Fig. 4C). For KEGG enrichment analysis, the top20 enrichment pathways of DE-mRNA was shown in Fig. 4B, among them, pathways involved in reproductive hormone synthesis and secretion including oxytocin signaling, aldosterone synthesis, and secretion, insulin secretion, thyroid hormone synthesis, and GnRH signaling, as well as photoperiodic response pathway like Circadian entrainment. Moreover, the top20 enrichment pathways of DE-lncRNA targets showed that two reproduction associated pathways including Hippo and MAPK were significantly enriched (Fig. 4D).

### Screening of potential reproduction-related lncRNAs in ovine PD

**Figure 5:**
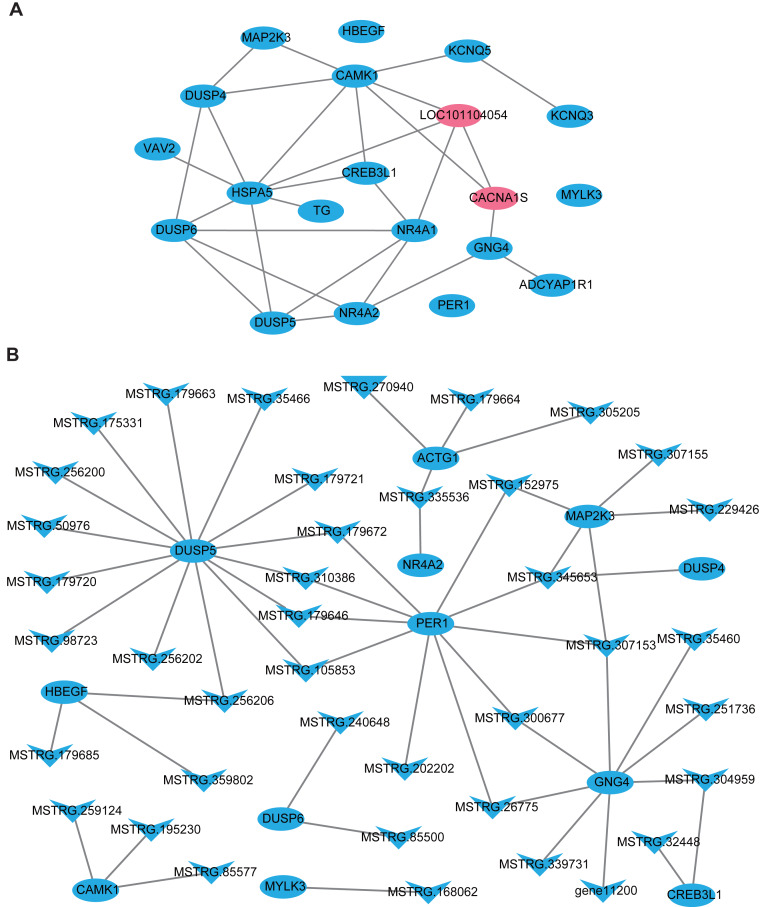
The network of differentially expressed transcripts involved in reproduction and photoperiodic response in the PD. (A) The network of 20 DE-mRNAs involved in reproduction and photoperiodic response in the PD. (B) The network between DE-mRNAs with DE-lncRNAs involved in reproduction and photoperiodic response in the PD. Circles and “V” represent mRNAs and lncRNAs, purple and blue represent up-regulated and down-regulated transcripts respectively.

In this part, firstly, 109 DE-lncRNA including 43 up-regulated and 66 down-regulated lncRNAs, as well as 93 DE-mRNA including 4 up-regulated and 89 down-regulated mRNAs were selected to construct the overall differentially expressed transcripts network (Fig. S1). The network showed that lncRNA was co-expressed with multiple protein-coding genes, which indicates mutual regulation of lncRNA and mRNA in the PD. Subsequently, a total of 20 DE-mRNAs, such as *MAP2K3, TG, NR4A1, CREB3L1, CACNA1S*, *ADCYAP1R1, PER1,* and a new gene *LOC101104054*, related to reproduction and photoperiodic response were used to construct the mRNA-mRNA network according to the KEGG enrichment results (Fig. 5A). To obtain the lncRNAs which participate in the ovine photoperiod induced reproductive activities, 40 lncRNAs, and its 11 targeted DE-mRNA which were discovered in the above mRNA-mRNA network were used to construct the interaction network. Surprisingly, the expression of these lncRNAs and mRNAs involved in sheep reproduction were all down-regulated in the PD (Fig. 5B). These findings suggest that PD tissue is greatly sensitive to in photoperiodic regulation of sheep reproduction.

## Discussion

The PD of pituitary is a crucial functional endocrine organ in the HPG axis that regulates mammalian onset of puberty and reproductive seasonality through the synthesis and secretion of reproductive hormones including FSH, LH and PRL. Recently studies have shown that synthesis and secretion of pituitary gonadotropins were regulated in the transcriptional level, in which noncoding RNA plays an important role (Yin et al., 2015; Lu et al., 2018; Pickard & Williams, 2016). Growing pieces of evidence indicate the important roles of lncRNAs in animal reproduction with the maturity of sequencing technology. For example, *Liu* found that several lncRNAs were involved in the reproductive related pathways such as TGF-*β* and PI3K-Akt by targeting the genes in the ovaries of Duroc pig (Liu et al., 2018), moreover, similar findings also exist in sheep (Feng et al., 2018; Wang et al., 2018), goat (Emanuele et al., 2018), chicken (Liu et al., 2018b) and other special economic animals (Chen et al., 2017; Sohel et al., 2013). In sheep, the lncRNAs in several tissues were related to animal reproductive or estrous activities (La et al., 2019; Zhang et al., 2019; Zheng et al., 2019; Feng et al., 2018). To better understand the effect of photoperiod on pituitary function, we established the OVX+E_2_ sheep model which has been frequently used to study the response to photoperiod and reproductive endocrine changes in seasonal reproduction animals (Legan et al., 2015; Scotti, Place & Demas, 2007; Jackson et al., 2013), subsequently, genome-wide analyses to identify differentially expressed mRNAs and lncRNAs in the pituitary of the above OVX+E_2_ sheep which treated with different photoperiod. The DE-lncRNAs and DE-mRNAs were used to reveal their functions in ovine pituitary, therefore this research provides a valuable resource for further studies of functional lncRNAs in the sheep pituitary.

Previous studies have proved that lncRNA was located in the protein-coding gene and can target this gene to play a regulatory role (Ulitsky et al., 2011; Guttman & Rinn, 2012). In this study, 48308 lncRNAs and 19906 mRNAs were identified in the pituitary of Sunite ewes, and the more lncRNAs were found compared with the earlier studies in the ovine pituitary (Zheng et al., 2019; Li et al., 2019b), suggesting that photoperiod may induce the production of more lncRNAs which participate in the regulation of pituitary function. The functional annotation of DE-lncRNA and DE-mRNA in case and control can reveal their roles clearly in a particular trait. In the present study, 20 of the DE-mRNAs (Fig. 5A), including *MAP2K3*, *TG*, *NR4A1*, *CREB3L1*, *CACNA1S* and *ADCYAP1R1*, were enriched in the pathways which were involved in the hormone synthesis and release, such as aldosterone synthesis and secretion, insulin secretion, thyroid hormone synthesis (Fig. 4B), and these pathways have been demonstrated to be involved in the mammalian reproductive regulation (La et al., 2019; Zheng et al., 2019; Li et al., 2019b). More interestingly, *ADCYAP1R1* and *PER1* were enriched in photoperiodic change-related pathway circadian entrainment. *ADCYAP1R1* was the receptor gene of pituitary adenylate cyclase-activating polypeptide type 1 receptor (*PAC1*), *Mercer et al.* found that SNP rs2267735 within the *ADCYAP1R1* located within a predicted estrogen response element, which regulates gene transcription when bound to estradiol (E_2_) activated estrogen receptor alpha (ER *α*) (Mercer et al., 2016). However, numerous pieces of evidence have confirmed that the concentration of E_2_was strongly affected by seasonal or photoperiodic change (Lai et al., 2012; Abdul-Rahman et al., 2016; Carr et al., 2003). *PER1* is one of the typical clock genes, whose expression is significantly correlated with circadian rhythm (Albrecht & Eichele, 2003; Messager et al., 2001). Our findings indicated that photoperiod can change animal reproduction state through transcriptional regulation of key pathways in the mRNA level.

LncRNA can regulate the transcriptional activity of target genes and participate in organ function. In this study, we firstly constructed all the DElncRNA-DE mRNA interaction networks with the photoperiod change (Fig. S1), which shown that lncRNAs can regulate the expression of target genes through its up-down regulation. To more accurately search for the reproduction-related lncRNAs, we constructed the interactions of lncRNAs-mRNA according to the DE-mRNA related to reproduction which described above. Surprisingly, all of these transcripts were down-regulation. In general, SP stimulus induces secretion of thyrotropin (TSH) from the pars tuberalis (PT) of the anterior pituitary gland and this PT-derived TSH locally activates thyroid hormone (T3) within the hypothalamus, which in turn induces gonadotropin-releasing hormone (GnRH) and then gonadotropin secretion (Nakayama & Yoshimura, 2017). However, *CREB3L1* can mediate functional and structural adaptation of the secretory pathway of thyroid hormone (García et al., 2017), MSTRG.32448 and MSTRG.304959 targeted *CREB3L1* may be involved in the ovine reproduction by regulating the secretion of thyroid hormone. MAPK signaling has been reported to be associated with reproductive activity in sheep (Zheng et al., 2019), goat and rat (Gao et al., 2018), as well as there is accumulating evidence that extracellular signal-regulated kinase (ERK) is one of the mitogen-activated protein kinases (MAPKs), two ERK specific phosphatases DUSP5 and DUSP6, which have been demonstrated to be markers of MAPK signaling activation (Buffet et al., 2017). Moreover, the up-regulation of DUSP6 regulates the duration of ERK activation (Higa et al., 2018). This study suggested that lncRNA MSTRG.240648 and MSTRG.85500 may participate in the MAPK signaling activation through targeting *DUSP6*. Besides, 10 lncRNAs targeting *PER1* gene, may relate to the photoperiod change, but the specific functions are still need systematic research.

## Conclusion

In conclusion, transcriptome analyses are useful methods to understand the effects of photoperiodic change on seasonal reproduction in ewes. In this study, our differentially expressed analysis identified pivotal reproductive genes and lncRNAs in the pituitary, as well as their interaction relationship based on the OVX+E_2_ ewes. Function annotation analysis indicated that the reproductive hormone and photoperiod response-related pathways including oxytocin signaling, aldosterone synthesis and secretion, insulin secretion, thyroid hormone synthesis, and circadian entrainment were enriched in the pituitary. Several lncRNAs such as MSTRG.240648, MSTRG.85500, MSTRG.32448 and MSTRG.304959 targeted *CREB3L1* and *DUSP6* may be involved in the ovine reproductive activities. Besides, 10 lncRNAs may relate to the photoperiod change because of targeting *PER1* gene. These findings in transcriptome provide a valuable resource for reproduction-related transcripts, and the interactions between DE-lncRNAs, DE-mRNAs and the enriched pathways provide clues for further study on the role of the pituitary regulated seasonal reproductive in ewes.

##  Supplemental Information

10.7717/peerj.10953/supp-1 Supplemental Information 1 qPCR data

10.7717/peerj.10953/supp-2 Supplemental Information 2 Supplemental figures and tables **Figure S1** The network between DE-mRNAs with DE-lncRNAs in the pituitary. Circles and “V” represent mRNAs and lncRNAs, line represent interaction between lncRNAs and mRNAs. Red and green represent up-regulated and down-regulated transcripts respectively.
**Table S1**Differentially expressed mRNA between the SP42 with LP42.
**Table S2**Differentially expressed lncRNA between the SP42 with LP42.
**Table S3**Top30 GO terms of differentially expressed mRNA between the SP42 with LP42.
**Table S4**Top30 GO terms of differentially expressed lncRNA targets between the SP42 with LP42.
**Table S5**Top20 differentially expressed mRNAs enriched KEGG pathways between the SP42 with LP42.
**Table S6**Top20 differentially expressed lncRNA targets enriched KEGG pathways between the SP42 with LP42.

## Additional Information and Declarations

### Data Availability

The following information was supplied regarding data availability: RNA-seq data are available in the NCBI Sequence Read Archive repository: PRJNA600124 (accession number SRP241089).
