# Supplementary figures and images for: Photoperiod induced the pituitary differential regulation of lncRNAs and mRNAs related to reproduction in sheep
Source: PeerJ. 2021 Apr 21;9:e10953. doi: 10.7717/peerj.10953 (PMC8067910; doi:10.7717/peerj.10953)

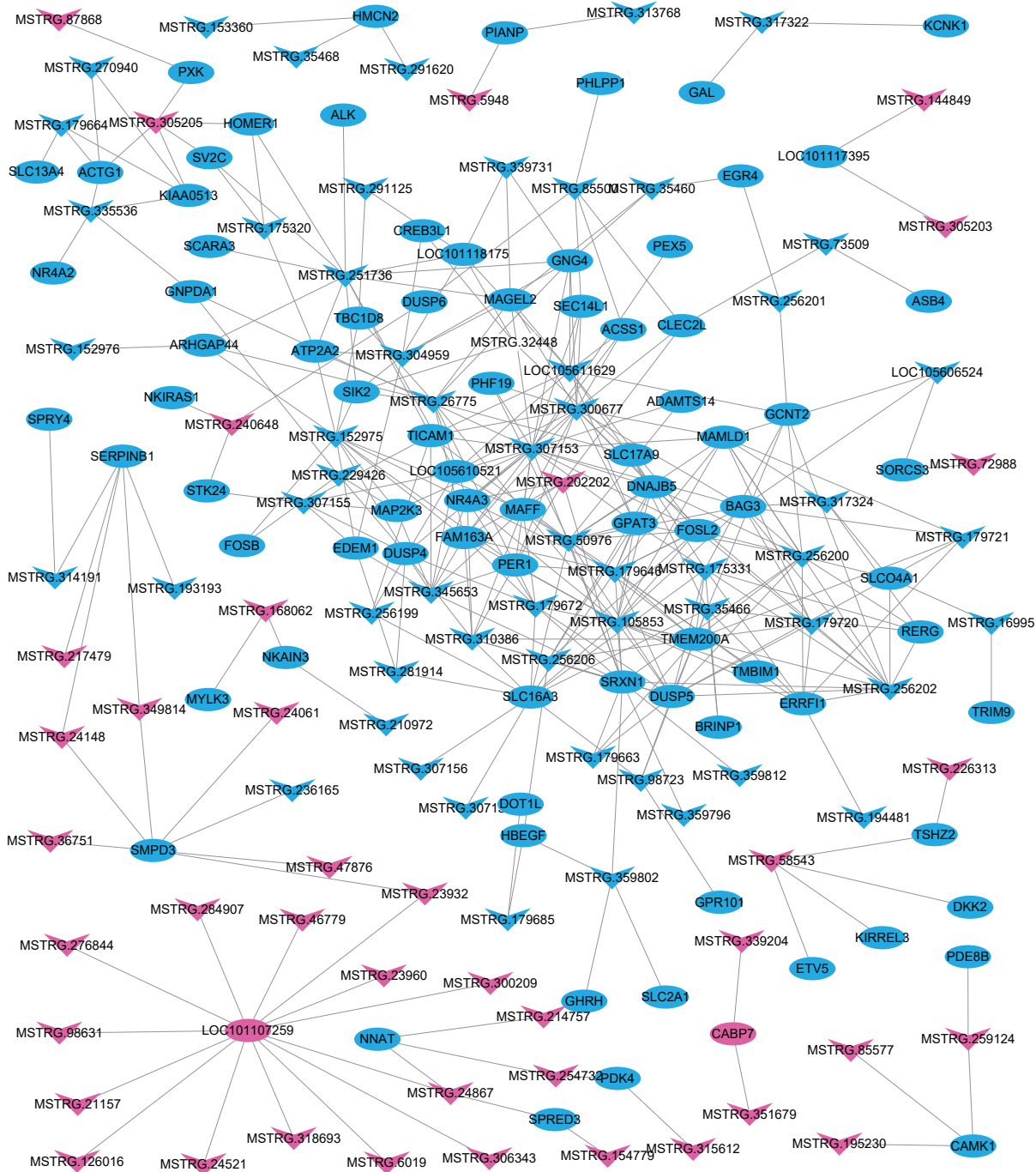

Supplement: Supplemental Information 2 — Figure S1 The network between DE-mRNAs with DE-lncRNAs in the pituitary. Circles and “V” represent mRNAs and lncRNAs, line represent interaction between lncRNAs and mRNAs. Red and green represent up-regulated and down-regulated transcripts respectively. Table S1Differentially expressed mRNA between the SP42 with LP42. Table S2Differentially expressed lncRNA between the SP42 with LP42. Table S3Top30 GO terms of differentially expressed mRNA between the SP42 with LP42. Table S4Top30 GO terms of differentially expressed lncRNA targets between the SP42 with LP42. Table S5Top20 differentially expressed mRNAs enriched KEGG pathways between the SP42 with LP42. Table S6Top20 differentially expressed lncRNA targets enriched KEGG pathways between the SP42 with LP42. [file peerj-09-10953-s002.zip › Supplementary materials/figure S1.pdf]
